# Novel genetic assessments for cancer patients: where does medical imaging stand in the future of personalised medicine?

**DOI:** 10.1002/jmrs.762

**Published:** 2024-02-15

**Authors:** Sally L. Ayesa

**Affiliations:** ^1^ School of Medicine University of Sydney Camperdown New South Wales Australia; ^2^ Department of Nuclear Medicine Royal North Shore Hospital St Leonards New South Wales Australia; ^3^ Department of Medical Imaging & Nuclear Medicine Gosford & Wyong Hospitals Gosford New South Wales Australia

## Abstract

As our understanding of genetics in cancer care improves, the role of personalised medicine for patients continues to grow. With the increasing emergence of novel technologies for patient assessment, such as the evaluation of circulating tumour DNA, we must reflect on the potentially changing role that medical imaging will play in the future of optimal patient care.

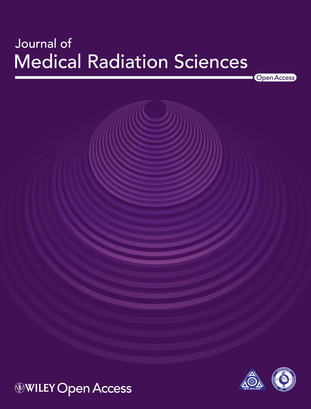

‘Personalised medicine’ has been a much‐discussed topic within medical imaging circles, even prompting the authorship of White Papers from the European Society of Radiology and discussion papers across professional journals.^1^, ^2^ With our ever‐growing understanding of the role that tumour genetics and a patient's DNA play in disease, we are increasingly looking to provide individualised treatments to our patients in the hope of improving prognosis.

This is most evident in the management of cancer patients, where the genetic characteristics of a tumour often play a role in the selection of the most appropriate therapy. A patient with non‐small cell lung cancer with an epidermal growth factor receptor (EGFR) mutation now has access to targeted therapies such as tyrosine kinase inhibitors, which disrupt intracellular pathways within cancer cells. A patient diagnosed with well‐differentiated metastatic prostate cancer now may have the option of receiving lutetium‐177 (Lu177) prostate‐specific membrane antigen (PSMA) therapy, which delivers targeted radiotherapy to sites of disease at a molecular level. As we seek to fight cancers at the cell, medical imaging remains as important as ever, and we as a specialty need to be across how the differences in technology and treatment will impact how we image these patients and interpret the results of relevant studies.

The article by Post and colleagues^3^ published in this month's edition of the journal explores just this, considering how emerging diagnostic options can complement the existing standard of care for patients with HPV‐driven oropharyngeal cancer. For their cohort of patients, the authors assessed how the results of fluorine‐18‐fluorodeoxyglucose positron emission tomography with low dose computed tomography (F18‐FDG PET/CT) following neck‐dissection correlated with the more novel diagnostic assessment of circulating tumour DNA (ctDNA).

The evaluation of ctDNA has grown from the decade‐old discovery of circulating cell‐free nucleic acid in the blood originating from a range of clinical conditions, but most importantly malignancy. The hostile cellular environment produced by a solid malignant tumour results in higher levels of necrosis and apoptosis within neoplastic tissues, with the dead cells engulfed by macrophages and broken down. The macrophages release fragments of DNA and messenger ribonucleic acid (mRNA) into body fluids, which can be isolated and analysed with cutting‐edge genetic testing.^4^ Circulating tumour cells (CTCs) are cancer cells that have been shed into the blood by a malignant tumour, the eventual cause of disseminated metastatic disease,^5^ as well as direct secretion of smaller fragments of DNA and mRNA from cells within the primary solid tumour, have also been hypothesised to contribute to circulating free nucleic acid.^4^

Traditionally, histopathological analysis of tumour has been limited to samples of the physical tumour itself obtained via resection or biopsy sampling of the lesion. Procedural radiology plays a key role in this sampling for many patients, with imaging‐guided biopsy under CT, ultrasound, fluoroscopy or even magnetic resonance imaging guidance being able to offer patients minimally invasive procedures with inherently lower risk of complications and reduced morbidity compared to open approaches. In current standard practice, tumours are staged and monitored by a combination of clinical, biochemical and imaging assessment, considering the underlying histopathology of the cancer and its genetics. Unfortunately, our current approach is limited by its inability to detect the earliest stages of microscopic spread or recurrence of tumour, which can go undetected.^6^ Moreover, the cell populations in malignant tumours are heterogenous and ever evolving, meaning that there is always the risk the prescribed therapy may not be effective for an entirety of a patient's cancer burden, or that genetic characteristics may change within a line of cells rendering them no longer susceptible to the current treatment.^5^

What ctDNA, microRNA and CTC analyses hope to provide is an even less invasive approach than percutaneous biopsy, offering a ‘liquid biopsy’ option based on the analysis of biological fluids. The utility of CTCs and ctDNA has been most extensively evaluated in peripheral blood, but researchers have also considered other body fluids including cerebrospinal fluid, saliva, urine, bile, and peritoneal or pleural fluid.^7^

Circulating tumour cells and ctDNA evaluation in cancer patients has been described as having the potential to impact patient care in several ways, including for early detection of disease and patients on current treatments or in the post‐treatment monitoring phase. Given the greater potential for positive clinical outcomes with early detection of aggressive cancer, there is hope that the research into CTCs and ctDNA will eventually find its way into improving the initial diagnostic evaluation of oncology patients by risk stratifying aggressive versus more indolent disease.^8^ For patients on treatment or post‐treatment, there is the potential for CTC and ctDNA testing to give greater insight into the behaviour of a patient's individual disease at a molecular and genetic level, ultimately playing a role in determining whether to continue therapy, alter the dosage or intensity of treatment, add another agent in the event of new mutated cell lines or cease therapy altogether if it is seen to be futile.^5^ But there is work to be done before the technology reaches daily practice.

As technologies for the detection of ctDNA, circulating microRNA and CTCs improve, interest in the field, and the volume and quality of research grows.^7^ With this, translational research becomes more important and we as clinicians will increasingly consider the place that these analyses could have within clinical practice. The specifics of just how these advancements will be integrated into standard patient care remain uncertain, however.

Too often in medicine, our specialties exist across silos of practice despite the general understanding that collaboration yields benefit to patient care and innovation of practice. In medical imaging we are comfortable with the constantly evolving landscape of our sphere of practice – inherently linked with improved scanner technologies and software applications, and the emergence of artificial intelligence – but we also need to be aware of the developments outside of the radiology and nuclear medicine department.

Medical imaging professionals have been experts at delivering personalised medicine for as long as medical imaging as a specialty has existed, by advocating for and delivering unique imaging plans depending on the patient's individual presentation and clinical needs.^2^ Moving forward, the specific nature of the role we play will inevitably change with technological progress. As we as researchers and clinicians further understand the cellular and molecular drivers of disease, this will be followed by novel investigative techniques that better assess and manage the inherently complex molecular environment encountered in cancer. Medical imaging departments will ultimately need to adapt to accommodate changing infrastructure, financial considerations and administrative needs to continue to provide a high standard of care.^1^

While the future of our specialty is of course uncertain, it is also exciting. The onus is on us as medical imaging professionals to consider the evolving nature of our role in complex care, and how best we can engage with our colleagues and patients to deliver the pinnacle of personalised medicine.

## Funding information

The author declares the financial support of Siemens Healthineers (educational speaker fees). She also declares that her additional research interests are supported by a grant from the Royal Australian and New Zealand College of Radiologists (not related to the development of this manuscript).

## Conflict of Interest

The author declares no conflict of interest.

## Data Availability

No new data has been collected during the course of preparing this manuscript.
